# Host–Microbiome Interaction in the Intensive Care Unit

**DOI:** 10.3390/diseases13080250

**Published:** 2025-08-07

**Authors:** Maria Adriana Neag, Andrei Otto Mitre, Irina Georgiana Pomana, Maria Amalia Velescu, Claudia Militaru, Georgiana Nagy, Carmen Stanca Melincovici

**Affiliations:** 1 Department of Pharmacology, Toxicology and Clinical Pharmacology, Iuliu Hatieganu University of Medicine and Pharmacy, 400337 Cluj-Napoca, Romania; maria.neag@umfcluj.ro (M.A.N.); claudia.militaru@umfcluj.ro (C.M.); 2 Department of Morpho-Functional Sciences, Discipline of Pathophysiology, Iuliu Haţieganu University of Medicine and Pharmacy, 400012 Cluj-Napoca, Romania; 3 Anaesthesia and Intensive Care Unit, “Prof. Dr. Octavian Fodor” Regional Institute of Gastroenterology and Hepatology, 400162 Cluj-Napoca, Romania; irina.geor.pomana@elearn.umfcluj.ro; 4 Cardiology Department, Clinical Rehabilitation Hospital, 400347 Cluj-Napoca, Romania; velescu.mariaamalia@elearn.umfcluj.ro; 5 Department of Internal Medicine, Iuliu Hatieganu University of Medicine and Pharmacy, 400006 Cluj-Napoca, Romania; georgiana.nagy@umfcluj.ro; 6 Department of Morpho-Functional Sciences, Discipline of Histology, Iuliu Hațieganu University of Medicine and Pharmacy, 400012 Cluj-Napoca, Romania; carmen.melincovici@umfcluj.ro

**Keywords:** gut microbiota, intensive care unit, critically ill patients, dysbiosis

## Abstract

Critical illness profoundly disrupts the gut microbiota leading to a state of dysbiosis characterized by reduced microbial diversity and overrepresentation of pathogenic taxa such as Enterobacteriaceae and Proteobacteria. This dysbiotic shift compromises gut barrier integrity and modulates immune responses, contributing to systemic inflammation and increasing susceptibility to nosocomial infections and multi-organ dysfunction. Nutritional strategies in the ICU significantly influence the composition and function of the gut microbiota. Enteral nutrition supports the maintenance of microbial diversity and gut mucosal health, whereas parenteral nutrition is associated with mucosal atrophy and further microbial imbalance. Emerging interventions, including the administration of probiotics, prebiotics, synbiotics, and fermented products like kefir, show promise in restoring microbial equilibrium and improving patient outcomes. This review presents current evidence on the alterations of the gut microbiota in critically ill patients, explores the systemic consequences of dysbiosis, and evaluates the impact of nutritional and microbiota-targeted therapies in improving patient outcomes.

## 1. Introduction

The human microbiota, consisting of diverse microbial communities inhabiting various sites throughout the human body, is fundamental to health maintenance and disease prevention. Recent research has highlighted its involvement in modulating immune responses, regulating metabolic functions, and providing protection against pathogenic colonization. Alterations in microbiota composition, or dysbiosis, are involved in numerous disorders ranging from infections to chronic inflammatory diseases [1,2].

Critically ill patients in intensive care units (ICUs) experience profound alterations in their microbiota, driven by both the pathophysiology of critical illness and the nature of intensive medical interventions. The use of broad-spectrum antibiotics, mechanical ventilation, invasive medical devices, and specialized nutritional support can significantly reduce microbial diversity. This reduction facilitates the overgrowth of opportunistic pathogens, thereby increasing the risk of secondary infections and contributing to organ dysfunction [3,4].

The dynamic interplay between the microbiota and distant organs—mediated through gut–organ axes—has received increasing attention. The gut–lung axis, for instance, influences pulmonary immunity and inflammatory responses, which is particularly relevant in ICU patients who are highly susceptible to respiratory complications. Similarly, the gut–brain axis is involved in neuroinflammatory and neurodegenerative pathways, while the gut–heart axis plays a role in cardiovascular function and pathology [5,6,7]. These systemic interactions underscore the importance of maintaining microbiota homeostasis, especially in critically ill individuals.

This review aims to synthesize recent findings on microbiota alterations in ICU patients, with a focus on how critical care interventions impact microbial communities and their systemic consequences. In addition, we explore emerging therapeutic strategies that target microbiota modulation to support recovery and improve clinical outcomes in this high-risk population, highlighting directions for future research.

## 2. The Microbiota—Pathways and Composition

### 2.1. Microbiome Composition: The Bacterial Taxa Abundance and Their Roles

**Figure 1:**
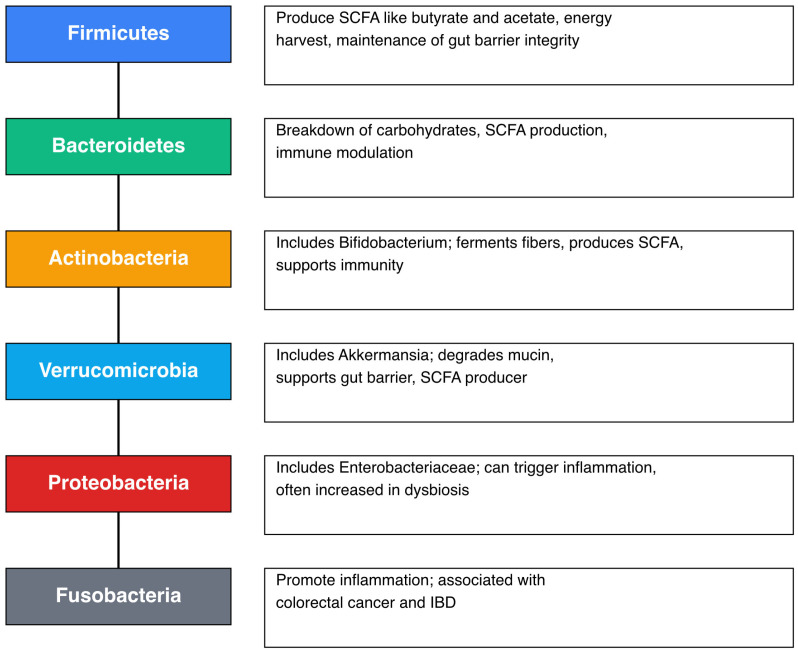
Microbiome composition and their roles.

The human gut microbiota is primarily composed of a few dominant bacterial phyla, with numerous other taxa present in lower abundance (Figure 1). These microbial groups contribute to health and disease through their metabolic products and functional activities. Among the most abundant phyla are *Firmicutes* and *Bacteroidetes*, which play central roles in maintaining gut homeostasis and modulating host physiology.

The Bacteroides species specialize in breaking down a wide range of plant-derived polysaccharides and sugar derivatives, converting them into fermentation products such as acetate and propionate [8]. These short-chain fatty acids (SCFAs) are essential for colonocyte nutrition, energy metabolism, and the maintenance of intestinal health. In addition to their metabolic functions, *Bacteroides* species contribute to colonization resistance by occupying ecological niches within the gut and producing antimicrobial compounds that inhibit the growth of pathogenic bacteria, including *Clostridioides difficile* [8,9,10].

B. Fragilis from the bacteroides phyla has a special immunomodulatory function. It produces capsular polysaccharide A that is a molecule known to stimulate and regulate immune system development, promote immune tolerance, and suppress excessive inflammation [9].

The Firmicutes hold a key role in fermentation and producing SCFAs (butyrate, acetate, propionate). Butyrate is particularly vital as it nourishes colonocytes, supports gut barrier integrity, and has anti-inflammatory properties. SCFAs also help regulate immune responses, reduce chronic inflammation, and support the development of immune tolerance [10,11].

In moderate abundance is the Actinobacteria phylum, with an important representative, Bifidobacterium. It contributes to the digestion of complex carbohydrates and dietary fibers, producing SCFA such as acetate and lactate, which support colonic epithelial health and metabolic regulation. Additionally, it exerts antimicrobial effects by competitively inhibiting pathogenic bacteria and reinforcing gut barrier integrity, thereby reducing systemic toxin exposure and infection risk. *Bifidobacterium* also synthesizes B vitamins and bioactive compounds, while modulating immune responses by promoting regulatory T cells, thereby reducing inflammation and supporting immune balance [12].

Although low in abundance, certain microbial taxa in the human microbiota serve as keystone species, exerting a disproportionately large influence on community stability and host health. These organisms contribute to pathogen resistance by competing for nutrients, producing inhibitory metabolites, and reinforcing gut barrier integrity, thereby preventing pathogen colonization and invasion [13].

Proteobacteria and Fusobacteria influence human health through their roles in the gut microbiome, often contributing to inflammation and disease. Elevated levels of Proteobacteria—particularly opportunistic pathogens like *E. coli*—are a hallmark of dysbiosis and can trigger pro-inflammatory immune responses. However, some species, such as *Alcaligenes*, support immune tolerance. Fusobacteria, especially *F. nucleatum*, are linked to colorectal cancer and inflammatory bowel disease. They promote inflammation, disrupt gut barrier function, and may impair immune recovery, although some strains produce beneficial metabolites like butyrate. Overall, both phyla are closely associated with gut dysregulation and disease progression [14].

### 2.2. Current Methods to Identify and Quantify the Microbiome

**Figure 2:**
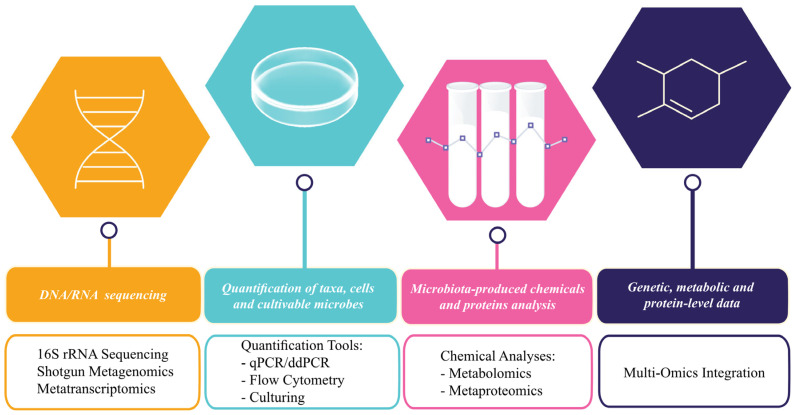
Methods to identify and quantify the microbiome.

Current methods to identify and quantify the microbiome primarily rely on advanced sequencing technologies combined with bioinformatics and statistical analysis tools (Figure 2).

The primary sequencing-based methods include:

16S rRNA Gene Sequencing

This targeted and cost-effective technique amplifies conserved and variable regions of the 16S rRNA gene to characterize bacterial communities, typically at the genus level. It is widely used for comparative analyses of microbial diversity and relative abundance across samples [15]. However, its limitations include low taxonomic resolution—unable to consistently resolve species or strains—exclusion of non-bacterial taxa (e.g., fungi, viruses), and susceptibility to PCR biases and primer selection effects [15].

Shotgun Metagenomic Sequencing

This untargeted approach sequences all DNA in a sample, enabling comprehensive identification of bacteria, archaea, viruses, and fungi, as well as functional genes related to metabolism, antibiotic resistance, and virulence [16]. It offers higher taxonomic resolution and functional insights than marker-based methods but is more costly, computationally intensive, and sensitive to host DNA contamination [17].

Metatranscriptomics

By analyzing microbial RNA, metatranscriptomics captures gene expression profiles, providing a snapshot of microbial activity and metabolic functions under specific conditions [18]. While this approach reveals dynamic microbial responses and low-abundance active taxa, it requires meticulous RNA preservation, is vulnerable to contamination, and remains limited by incomplete transcript annotation in current reference databases [19].

To address limitations of relative abundance estimates and further characterize microbial communities, additional quantification and functional methods have been developed:

Quantitative PCR (qPCR) and Digital Droplet PCR (ddPCR)

These highly sensitive approaches quantify total bacterial load or specific taxa by targeting conserved genes. Although efficient, they can introduce amplification bias and are limited to predefined targets [20,21].

Flow Cytometry

This technique enables direct microbial cell counting and discrimination between live and dead cells using viability dyes. It requires extensive sample preparation and may not accurately detect all microbial groups [20,21].

Culture-Based Methods

Traditional culturing allows for the enumeration of viable microbes through colony-forming units (CFUs), but it is limited to cultivable species and often underestimates community diversity [20,21].

Chemical Analysis

Metabolomics uses mass spectrometry or nuclear magnetic resonance (NMR) to detect microbial metabolites such as short-chain fatty acids, bile acids, and vitamins, which influence host physiology, immunity, and gut barrier integrity [14].

Metaproteomics employs mass spectrometry to identify and quantify microbial proteins, revealing functional activity within microbial communities [14].

Multi-Omics Integration

Combining genomic, transcriptomic, proteomic, metabolomic, and epigenomic data provides a systems-level view of microbiome–host interactions, enabling deeper insights into microbial function and disease mechanisms [22].

Collectively, these methods illustrate the complex role of the gut microbiome in regulating nutrient metabolism, immune function, and inflammatory pathways. With the growing availability of integrated and functional microbiome analyses, the gut–organ axes (e.g., gut–liver, gut–lung, gut–kidney, gut–brain, gut–heart) represent promising targets for microbiota-based interventions in critical illness. These connections will be further explored in the following sections.

### 2.3. The Microbiota-Involved Pathways and Axis

**Figure 3:**
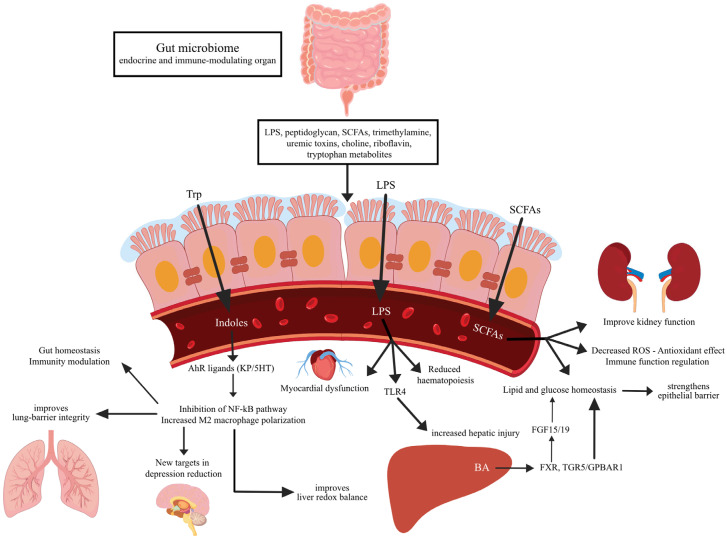
Microbiota-related pathways and interaction with other organs.

The gut microbiome functions as an endocrine and immune-modulating organ exerting systemic effects on distant organs (Figure 3). These effects are mediated through the production and release of microbe-associated molecular patterns (MAMPs), such as lipopolysaccharide (LPS) and peptidoglycan, as well as a wide range of microbial metabolites—including short-chain fatty acids (SCFAs), trimethylamine, uremic toxins, choline, tryptophan-derived compounds, and riboflavin [23,24]. Disruption of the intestinal barrier can lead to translocation of LPS into the systemic circulation, which contributes to hepatic injury through Toll-like receptor 4 (TLR4) activation and the subsequent inflammatory cascade [25]. In cirrhosis, increased intestinal permeability is associated with elevated levels of pro-inflammatory cytokines and systemic inflammation, further exacerbating disease progression [26,27].

Because ICU patients present with dysbiosis, it is important to understand the major gut–organ axes and their possible involvement in critical care conditions [28]. In this section, we will discuss the various pathophysiological interactions of the gut microbiome and its metabolites with the body.

Bile acids (BAs) are steroid derivatives synthesized from cholesterol in the liver and secreted into the intestine, where they facilitate the absorption of dietary fats and fat-soluble vitamins. Beyond their digestive role, BAs act as signaling molecules by activating nuclear and membrane receptors, including the farnesoid X receptor (FXR) and the Takeda G protein-coupled receptor 5 (TGR5, also known as GPBAR1), thereby influencing lipid and glucose metabolism [29,30,31]. BAs activate FXR that further regulates gene expression involved in lipid and glucose metabolism [32,33]. In a mouse model of diabetes, FXR activation lowers blood glucose and improves insulin sensitivity. It also inhibits hepatic gluconeogenesis, increases hepatic glycogen synthesis, and lowers triglyceride and cholesterol levels [34,35]. However, other studies on FXR-deficient obese mice had shown reduced adipose tissue mass and improved glucose homeostasis, effects that do not seem to be mediated by liver-FXR activity [36,37]. By altering the gut microbiota in a mice model of high-fat-diet-induced non-alcoholic fatty liver disease (NAFLD), FXR inhibition led to reduced triglyceride levels and liver lipid accumulation [38]. In obstructed bile flow models, administration of BAs and FXR activation preserved mucosal integrity and prevented intestinal damage [39]. FXR also activates the fibroblast growth factor 15 (rodents)/19 (non-rodents) (FGF15/19) pathway [32]. FGF15/19 is involved in hepatic fatty acid synthesis and lipid metabolism, reducing lipid levels and accumulation [40,41]. Different taxa and antibiotic treatments influence the levels of FGF15 and FXR, which can lead to metabolic abnormalities and intestinal barrier disruption [42,43]. In experimental cirrhosis, FXR can modulate the epithelial barrier and reduce bacterial translocation [44]. In skeletal muscles, FGF19 can potentially ameliorate atrophy and sarcopenia [45]. FXR-TGR5 leads to glucagon-like peptide 1 activation and normal gastrointestinal motility [46]. TGR5 further contributes to immune modulation by reducing pro-inflammatory cytokines in Kupffer cells while preserving anti-inflammatory cytokines and promoting M2 macrophage polarization [47,48].

SCFAs—including formate, acetate, propionate, and butyrate—are end-products of the microbial fermentation of non-digestible carbohydrates. These metabolites are vital for maintaining gut barrier integrity and mucosal health, but they also exert systemic effects on glucose and lipid metabolism, appetite regulation, and immune modulation [49,50]. Antibiotic-induced butyrate depletion in early life impairs natural killer cell maturation and IL-18 production, compromising immune development [51]. In models of hepatic sinusoidal obstruction, butyrate supplementation improved intestinal barrier integrity and reduced liver injury [52]. Butyrate treatment can also reduce reactive oxygen species levels and tissue injury via activating the Nrf2 antioxidant pathway [53]. In LPS-stimulated neutrophils, both butyrate and propionate can reduce the levels of TNF-α, nitric oxide, and NF-kB activation [54]. In sepsis-associated encephalopathy, fecal microbiota transplantation restored butyrate levels and reduced oxidative stress markers in the hippocampus, offering neuroprotective effects [55].

Tryptophan (Trp), an essential aromatic amino acid, is metabolized by gut microbiota into bioactive compounds including indoles, kynurenine (KP), and serotonin (5-HT). Indoles act as ligands for the aryl hydrocarbon receptor (AhR), a key regulator of immune function, intestinal homeostasis, and liver redox balance [56,57,58]. Reduced AhR activation has been associated with reduced innate immune cells and increased susceptibility to enteric infections [59]. In liver diseases, including models of liver fibrosis and inflammation, indole treatment via AhR activation reduced liver damage, the levels of pro-inflammatory cytokines, and oxidative stress [60,61,62,63]. In a nephrocalcinosis model, AhR activation shifted macrophage polarization towards the M2 phenotype, attenuating the renal damage [64]. Also, AhR activity is important in maintaining lung barrier integrity after viral infection, its loss being associated with increased lung damage and increased alveolar infiltration [65]. Additionally, the gut microbiota–tryptophan–5-HT axis has been proposed as a potential target in adolescent depression, given its role in modulating serotonin levels [66].

Related to other organ injuries, in LPS-induced lung injury, microbiota imbalances increase oxidative stress and inflammation via regulating the TLR4/NF-kB pathway. Fecal microbiota transplantation mitigated in part these effects, reducing lung injury and inflammation [67]. This could provide another strategy in reducing the effects of acute respiratory distress syndrome or acute lung injury [68]. In the context of acute kidney injury (AKI), gut dysbiosis exacerbates disease progression by decreasing SCFA production and increasing uremic toxins, thereby creating a feedback loop that worsens kidney function [69]. Similarly, in cardiac disease models, increased gut permeability and dysbiosis elevate systemic LPS, D-lactate, and inflammatory cytokine levels, contributing to myocardial remodeling and dysfunction [70,71]. Additionally, antibiotic-induced dysbiosis has been linked to impaired hematopoiesis, indicating a connection between the gut microbiome and bone marrow suppression [72].

## 3. Microbiota Alterations in the ICU Patient

### 3.1. Particularity of the ICU Patient

ICU patients are unique due to the severity of their illnesses. They often experience multiple organ dysfunction and require advanced medical and nursing care. Their complex medical conditions may necessitate interventions such as mechanical ventilation, invasive monitoring, and various forms of life support. Although these measures are essential, the intensity of treatment can have significant effects on the body and is often accompanied by side effects. The microbiome of ICU patients is profoundly affected by a range of factors, leading to decreased microbial diversity, a rise in pathogenic organisms, and increased risk of complications. The main factors influencing the microbiome in ICU patients are:

### 3.2. Critical Illness and Organ Dysfunction

The underlying critical illness itself, including severe organ pathology and multiple organ dysfunction, alters the microbiota by promoting inflammation and immune dysregulation [73]. Critical illness induces a rapid decline in the diversity of the gut and other microbiomes, favoring the dominance of pathogenic bacteria over beneficial commensals [74,75]. This dysbiosis is associated with increased susceptibility to hospital-acquired infections, sepsis, and further organ failure. There are physiological changes in critical illness: Decreased oral intake reduces the immigration of beneficial, food-associated microbes; intestinal dysmotility and decreased elimination allow pathogens to accumulate, while impaired mucosal integrity increases the translocation of bacteria and their products into the bloodstream, promoting systemic inflammation and distant organ dysfunction. For ICU patients, altered immune responses are also common. Decreased IgA and defensin production reduce the host’s ability to suppress pathogenic bacteria [76,77].

Organ dysfunction, such as acute kidney injury or brain dysfunction, may both result from and contribute to microbiome disruption. Gut stressors—nutrient deprivation, inflammation, and altered oxygen gradients—favor the overgrowth of Proteobacteria and depletion of beneficial anaerobes [76,78].

Notably, ICU patients cannot be regarded as a homogenous group. Emerging evidence suggests that different critical illnesses (e.g., sepsis, trauma, respiratory failure) result in distinct gut microbiota profiles—so-called “ICU-enterotypes.” These microbiota configurations, characterized by specific bacterial signatures (e.g., *Bacteroides*/Enterobacteriaceae or Enterococcus-dominant profiles), are independently associated with clinical outcomes such as septic shock or mortality risk [79].

While underlying disease strongly influences microbiome composition, the ICU environment (antibiotics, nutrition, inflammation) induces widespread dysbiosis common across patients. This means stratifying ICU patients by disease alone captures only part of the picture, as microbiome signatures provide additional clinical insights, including susceptibility to infection and immune dysfunction. ICU patients are best categorized both by their underlying disease types and by their distinct gut microbiota profiles. The disease classification provides clinical context, while microbiome-based categorization offers additional, independent insights into patient risk, immune status, and outcomes. Gut microbiota dysregulation should be viewed not just as a marker but as a potentially modifiable contributor to disease progression. Advances in ICU care are moving toward integrating these microbiome distinctions into routine patient stratification to enable more precise and effective interventions [79,80].

### 3.3. Patient-Specific Factors

Underlying diseases such as diabetes, cancer, and immunosuppression, along with an individual’s immune status, have a significant impact on the composition and function of the microbiome inflammation and barrier dysfunction [81,82]. Gut microbiota dysbiosis is present in both type 1 and type 2 diabetes. In these patients, there is a reduction in beneficial, butyrate-producing bacteria and an increase in microbial species that promote inflammation and metabolic dysfunction. Certain gut bacteria can produce metabolites, such as imidazole propionate, that impair insulin signaling and glucose tolerance, further exacerbating insulin resistance and metabolic complications [81,83].

Microbial dysbiosis can contribute to carcinogenesis by promoting chronic inflammation, producing genotoxic metabolites, and altering the local immune environment [84]. In cancer, dysbiosis contributes to carcinogenesis via chronic inflammation, genotoxic metabolite production, and immune evasion. For instance, Fusobacterium nucleatum suppresses natural killer cells and recruits immunosuppressive cell populations [85], whereas other microbes produce metabolites like inosine that enhance immunotherapy responses through T cell activation [82].

In immunosuppressed patients, loss of immune control allows opportunistic pathogens to dominate, impairing host defenses and reducing the efficacy of immune-based treatments [84,85,86].

The immune system and microbiome are in constant communication [83]. Changes in immune status can alter the gut environment, affecting which microbes thrive and which are suppressed, leading to either protective or harmful outcomes [84].

### 3.4. Antibiotic Use

Antibiotic usage is extremely common in the ICU, with prevalence rates exceeding 70% [87]. Regardless of their spectrum of activity, antibiotics are eliminating, indiscriminately, both beneficial and pathogenic bacteria, which facilitates colonization by drug-resistant and opportunistic pathogens [88]. The impact of antibiotics depends on factors such as the type and duration of antibiotic used, the number of previous courses, and the individual’s baseline microbiome health. Certain classes of antibiotics commonly used in the ICU determine a loss of microbial diversity and selection for organisms such as *Clostridioides difficile* and *Candida* spp. that can cause secondary infection [88,89,90].

Microbial diversity loss can occur rapidly, within days of initiation, and persist for months or even become permanent [87,91]. The disruption can have lasting consequences, including increased intestinal permeability, inflammation, reduced production of beneficial metabolites, such as SCFAs and altered immune cell development [92]. Antibiotic use also promotes the growth of antibiotic-resistant bacteria by eliminating susceptible strains, which can lead to long-term shifts in the microbiome composition and function [91].

### 3.5. ICU-Specific Medications

Critically ill patients are susceptible to further disruption of gut microbiota homeostasis and intestinal barrier integrity due to the administration of additional pharmacological agents. Medications commonly employed in the ICU setting are proton pump inhibitors, opioids, vasopressors, catecholamines, non-steroidal anti-inflammatory drugs (NSAIDs), and immunosuppressive therapies. All these therapies contribute to microbiome disturbances, either by altering gut pH, motility, or immune responses [90,93]. Proton pump inhibitors reduce gastric acid, allowing oral bacteria (*Rothia*, *Streptococcus*) to colonize the gut. This decreases microbial diversity and increases pathogenic genera like *Enterococcus* and *E. coli.* Dysbiosis promotes inflammation and compromises mucosal defense, raising susceptibility to infections like *C. difficile* [93,94].

Opioids are extensively utilized in the intensive care setting, primarily for the management of acute pain and sedation. Their use has been associated with significant alterations in gut microbiota composition, characterized by a reduction in beneficial genera such as *Lactobacillus* and *Bifidobacterium*, and an over-representation of opportunistic pathogens including *Staphylococcus* and *Enterobacter* spp. Furthermore, opioid-induced gastrointestinal hypomotility impairs the clearance of luminal bacteria, promoting microbial overgrowth. At the level of the intestinal barrier, opioids have been shown to disrupt tight junction integrity, leading to increased epithelial permeability. This facilitates bacterial translocation and systemic dissemination of microbial products such as lipopolysaccharides, contributing to systemic inflammation and potential neuroimmune dysregulation through the gut–brain axis [95].

Catecholamines (norepinephrine, epinephrine, and dopamine) are substances used in shock states to increase vasoconstriction and/or increase heart contractility. They promote the growth of harmful Gram-negative bacteria in the intestines through direct, non-nutritional mechanisms that exploit bacterial iron-scavenging systems and enhance virulence. They can act as chemical analogues to bacterial siderophores or stimulate endogenous siderophore production, promoting iron acquisition in the iron-depleted environment of the gut. This facilitates the proliferation of pathogens such as *Escherichia coli*, *Salmonella* spp., and *Pseudomonas aeruginosa* [96,97].

In addition to supporting growth, catecholamines enhance the expression of multiple virulent determinants. Norepinephrine, in particular, has been shown to upregulate genes encoding toxins such as Shiga toxins in enterohemorrhagic *E. coli* (EHEC) and the heat-labile toxin in enterotoxigenic *E. coli* (ETEC). Adhesion factors, including K99 pili, are also upregulated, promoting mucosal attachment. Moreover, catecholamines stimulate biofilm formation in pathogens like *E. coli*, which facilitates colonization, persistence, and increased resistance to antimicrobial agents [98,99]. The release of catecholamines during physiological stress has immunosuppressive effects that further support pathogen expansion. These include the downregulation of key mucosal immune components such as secretory immunoglobulin A and antimicrobial peptides [98,100]. They also contribute to intestinal dysbiosis by selectively favoring the growth of Gram-negative bacteria, especially members of the *Enterobacteriaceae* family, over beneficial commensal taxa. Norepinephrine inhibits the antimicrobial activity of probiotic bacteria, such as *Levilactobacillus* spp, further disrupting epithelial barrier integrity and promoting translocation of pathogenic organisms [97,99,100].

NSAIDs inhibit cyclooxygenase, reducing prostaglandins essential for mucosal health. This alters microbial composition, reducing diversity and promoting dysbiosis. Prostaglandin deficiency weakens mucosal integrity, increasing permeability and susceptibility to ulceration and inflammation [101].

Immunosuppressive therapies, particularly corticosteroids, significantly impact both the composition of the gut microbiota and the structural integrity of the intestinal barrier. Corticosteroid administration has been associated with a marked reduction in beneficial microbial taxa, with an anti-inflammatory effect, including *Faecalibacterium*, members of the *Ruminococcaceae* family, and *Bifidobacterium* spp. Concurrently, these therapies promote the overgrowth of opportunistic pathobionts, notably *Enterobacteriaceae* and *Enterococcus* spp., largely due to the suppression of immune-mediated microbial control. This shift in microbial balance not only reduces community diversity but also predisposes patients to secondary infections and systemic inflammatory responses [102]. The mechanisms underlying corticosteroid-induced dysbiosis include downregulation of antimicrobial peptides such as RegIIIβ and RegIIIγ, degradation of the mucus barrier due to reduced *Muc2* expression, and disruption of host circadian rhythms that are known to influence microbial community structure [102,103]. These factors collectively impair microbial regulation and contribute to a permissive environment for pathogenic overgrowth. In addition to microbiota alterations, corticosteroids directly impair gut barrier function. They inhibit myosin light chain kinase (MLCK), a key regulator of tight junction assembly, thereby compromising epithelial integrity and increasing intestinal permeability. Mucosal immune defenses are also attenuated, as evidenced by diminished secretory IgA coating of bacteria and impaired interleukin-22 signaling, both critical for maintaining barrier immunity [101].

### 3.6. Nutrition and Feeding Practices

Nutrition and feeding practices in ICU patients have a profound impact on the gut microbiome. Based on large-scale ICU data, approximately 6.2% of critically ill patients receive parenteral nutrition (PN) during their ICU stay, while about 15.6% receive enteral nutrition (EN) [103].

EN in the ICU significantly influences both the immediate and long-term recovery of the gut microbiome in critically ill patients with different effects on the gut microbiota based on the formulation.

Standard formulas (polymeric compounds) are the most used, suitable for patients with a functioning gastrointestinal tract. This consists of intact proteins, complex carbohydrates, and long-chain triglycerides [104].

Fiber-enriched formulas containing added soluble and/or insoluble fiber promote gut health and microbiota balance and are recommended for hemodynamically stable patients, particularly when diarrhea or GI tolerance is a concern [104,105]. In contrast, fiber-free formulas are indicated for patients at risk of bowel obstruction, severe dysmotility, or intolerance to fiber, such as in cases of ileus or recent abdominal surgery [104].

Disease-specific or therapeutic formulas are tailored to address specific clinical scenario such as high-protein for hypercatabolic states, low-electrolyte for renal impairment, glucose-controlled for diabetes, and immunonutrition formulas enriched with arginine, omega-3 fatty acids, antioxidants, or glutamine for patients with trauma, sepsis, or surgical interventions [106].

Peptide-based (semi-elemental) formulas, containing hydrolyzed proteins, medium-chain triglycerides, and simple carbohydrates, are used in patients with malabsorption syndromes or pancreatic insufficiency [104].

Elemental formulas, composed of free amino acids, simple sugars, and minimal fat, are reserved for severe digestive or absorptive dysfunction and are infrequently used in the ICU [107].

Plant-based enteral nutrition (PBEN), which emphasizes plant-derived proteins and fibers, are less commonly used but outperform traditional artificial formulations in restoring commensal microbiota following profound disruptions (antibiotic treatment or critical illness). PBEN helps replenish genera like Bacteroides and Phocaeicola, which are depleted during dysbiosis. Early data from pilot studies in humans, support that the plant-based approach leads to a more robust and varied gut microbiome, with increased resistance to colonization by potential pathogens [108,109].

Hospital-prepared formulas, containing mixtures of standard food ingredients, are used in settings with limited access to commercial formulas, though they present challenges related to nutrient consistency and sterility [110].

EN is generally more supportive of gut barrier integrity and microbial diversity compared to PN, which bypasses the gastrointestinal tract and can result in nutrient deprivation for commensal microbes, contributing to dysbiosis [111]. The absence of enteral stimulation is associated with a decline in beneficial microbial populations and increased colonization by pathogenic organisms, which further compromises epithelial barrier function and promotes systemic inflammation. However, it is important to recognize that exclusive enteral nutrition in the ICU does not fully replicate physiological feeding conditions [112,113].

Critically ill patients who receive total EN often show major alterations in their gut microbiota. The most abundant bacterial groups found in these patient intestines are Firmicutes, Proteobacteria, Bacteroidetes, Actinobacteria, and Verrucomicrobia. This overall composition differs from healthy controls, with a general trend toward reduced diversity and increased predominance of potentially harmful taxa. There is a marked increase in opportunistic and potentially pathogenic bacteria, notably genera such as Enterococcus and Klebsiella [114]. This overgrowth is particularly pronounced in patients with enteral nutrition-related diarrhea (END). In these cases, beneficial bacteria like Bacteroidetes and Subdoligranulum are significantly depleted, while Enterococcus and Klebsiella become dominant biomarkers of dysbiosis [112]. Taxa associated with gut health (Bacteroidetes or Subdoligranulum) decline in abundance. These bacteria are important, as they produce SCFAs that support gut barrier integrity and contribute to anti-inflammatory effects, nutrient absorption, and metabolic homeostasis [113].

The clinical consequences of dysbiosis are significant, particularly in the domains of immune competence and metabolic regulation. An overrepresentation of Enterobacteriaceae (*Klebsiella* and other nosocomial pathogens) has been associated with impaired innate immune responses, including diminished neutrophil activity. This immunosuppressed state increases susceptibility to hospital-acquired infections [115]. Furthermore, key metabolic pathways involved in nutrient utilization and immune modulation are profoundly disrupted in patients with severe dysbiosis, particularly those with END. These disruptions contribute to metabolic imbalances and persistent low-grade inflammation, which may exacerbate organ dysfunction and delay recovery [112,113].

### 3.7. Mechanical Ventilation, Invasive Monitoring, and ICU Environment

The intensive care environment, along with invasive medical interventions such as mechanical ventilation and catheterization, exerts a profound disruptive effect on the microbiome of critically ill patients, promoting dysbiosis and elevating the risk of nosocomial infections. Mechanical ventilation, in particular, is associated with a significant reduction in the alpha diversity of the respiratory microbiome, reflecting decreased species richness and evenness. Although the gut microbiome experiences similar changes, the effects tend to be less immediate and more gradual. The use of an endotracheal tube connects the oropharynx and lungs, facilitating the migration and colonization of potential respiratory pathogens such as *Pseudomonas aeruginosa* and *Gammaproteobacteria* in the lungs. During mechanical ventilation and the development of ventilator-associated pneumonia (VAP), the lung microbiome often becomes dominated by pathogenic bacteria, while beneficial commensals are diminishes [114,116,117]. Similarly, the use of intravascular and urinary catheters introduces foreign surfaces that serve as substrates for bacterial colonization and biofilm formation. These biofilms often harbor multidrug-resistant organisms and are highly resistant to both antimicrobial therapy and host immune responses. These infections can further perturb both local and systemic microbial ecosystems [118,119].

In addition, the ICU environment itself is a source of continuous microbial exposure. Patients are frequently in contact with contaminated surfaces, medical devices, and healthcare personnel, all of which contribute to the transmission of hospital-associated pathogens. This external microbial pressure often leads to colonization by exogenous organisms that displace the patient’s native microbiota, further exacerbating dysbiosis and compromising host defenses [114].

## 4. Microbiota Targeted Therapies

The human microbiota, called the “hidden organ”, contains over a hundred times more genetic information than the entire human genome. This “organ” consists of bacteria, yeasts, and viruses, present in different parts of the body (gut, skin, lungs, etc.). The main phyla of bacteria are Firmicutes, Bacteroidetes, Actinobacteria, Proteobacteria, Fusobacteria, and Verrucomicrobia, the first two being the majority types [120].

The human gut microbiota, this complex and dynamic microbial ecosystem, exerts major effects on host physiology. It plays a crucial role in nutrient metabolism, converting dietary substrates into bioactive metabolites such as SCFAs that act as key regulators of immune homeostasis and interact with the host’s immune, metabolic, and nervous systems. This microbial community also engages in bidirectional communication with distal organs, establishing integrated axes such as gut–brain, gut–liver, and gut–lung connections. Such interactions underscore the concept of the microbiota as a “metabolic organ” that influences systemic health. Dysbiosis has been causally linked to a wide range of pathologies, from those of the gastrointestinal system (e.g., inflammatory bowel diseases) to metabolic syndrome, neurodevelopmental disorders, and even cancer. Due to the importance of gut bacteria in the systemic homeostasis of the body, the gut microbiota is considered a “vital organ” [121].

Modifying the intestinal microbiome holds significant promise for decreasing the incidence and/or severity of numerous human diseases and conditions. The current challenge for the biomedical research community is to translate this growing understanding of the microbiome into effective medical treatments [122].

This understanding paves the way for innovative clinical strategies: live microbial therapies (prebiotics or next-generation probiotics), purified microbial metabolites (“post-biotics”), and microbiota-based adjuvants that can support existing treatments, such as immunotherapy or chronic disease pharmacology. Harnessing the microbiota for precise, disease-directed interventions appears to be a transformative frontier in medicine [123].

### 4.1. Probiotics

Probiotics are live microorganisms—usually bacteria or yeast—that provide health benefits to the host when consumed in sufficient quantities. When used as targeted therapy, they are carefully selected or engineered to interact specifically with certain physiological or pathological processes [124].

### 4.2. Prebiotics

Prebiotics are a non-digestible food component that supports the host’s health by specifically promoting the growth or activity of certain beneficial bacteria in the colon [125].

The key effects of this nutraceutical agents are outlined in Table 1.

### 4.3. Downsides of Microbiota Targeted Therapies in ICU Patients

The study by Elfiky et al. investigated whether the composition of the gut microbiome at ICU admission was associated with the risk of developing hospital-acquired infections (HAI). Comparing twenty ICU patients with thirty healthy subjects of the same age and sex, the researchers found a significant reduction in Firmicutes and a lower Firmicutes/Bacteroidetes ratio among ICU patients. Those who subsequently developed HAI showed a further depletion of beneficial species such as Bifidobacterium and Faecalibacterium prausnitzii, along with an increase in Lactobacilli. These microbial changes were negatively correlated with APACHE II scores, suggesting that early gut dysbiosis may predict a higher risk of infection and mortality. The study highlights the potential of microbiome modulation as a preventive tool in intensive care units [80].

Regarding to the role of probiotics in prevention of ventilator-associated pneumonia, the results are controversial. For example, in a study, Johnstone and collab. investigated whether the probiotic Lactobacillus rhamnosus GG could reduce the incidence of VAP in critically ill adults requiring mechanical ventilation. Patients in intensive care units were randomly assigned to receive either the probiotic or a placebo. Contrary to initial hypotheses, the study did not demonstrate a significant reduction in the incidence of VAP among those given the probiotic [138].

On the other hand, in a systematic review and network meta-analysis that included thirty-one randomized controlled trials with a total of 8339 ICU patients receiving mechanical ventilation, the results indicated that probiotic supplementation significantly lowers the risk of VAP, particularly in trauma patients. Mixed-strain probiotics and lower doses (<10^10^ CFU/day) were more effective than single-strain formulations or higher doses. However, despite these encouraging results, the overall quality of evidence was low, highlighting the need for further high-quality research to validate these conclusions [139].

The results emphasize the importance of judicious and evidence-based administration, rather than their routine use for the prevention of VAP in critically ill patients.

In a retrospective study, six cases of Bifidobacterium breve bacteremia associated with probiotic administration (strain BBG-01) were investigated in newborns admitted to the Japanese neonatal intensive care unit between June 2014 and February 2019. Among 298 infants who received probiotics, mainly premature or with congenital surgical conditions, the incidence of B. breve bacteremia was 2%, significantly higher than the previously reported national average. Most cases had underlying gastrointestinal pathologies, such as necrotizing enterocolitis, food protein-induced enterocolitis syndrome, ileus, or volvulus. Bacteremia was confirmed by positive blood cultures, and molecular analysis confirmed BBG-01 as the causative strain in five of six cases. The study highlights that although probiotic administration is generally safe and potentially beneficial for intestinal colonization and prevention of necrotizing enterocolitis, it may pose a risk of bacteremia in neonates with compromised intestinal integrity. Vigilance in the use of probiotics and extensive monitoring of blood cultures are recommended, especially in high-risk populations [140].

In a systematic review, Darbani et al. assessed the clinical efficacy of probiotic supplementation in reducing infections among hospitalized patients, encompassing both ICU and non-ICU populations. The review focused on randomized controlled trials (RCTs) that utilized various probiotic strains, evaluating their impact on the incidence of hospital-acquired infections, including VAP, Clostridioides difficile infection (CDI), bloodstream infections, and urinary tract infections. The results demonstrated a statistically significant reduction in several types of nosocomial infections, particularly VAP and CDI, among patients receiving probiotics. The authors propose several mechanisms underlying these protective effects (which we mentioned previously), such as modulation of the gut microbiota, strengthening of the intestinal barrier, inhibition of pathogen colonization, and immune system modulation. Nevertheless, they advise caution in interpreting the findings broadly, given the heterogeneity in probiotic formulations, dosages, treatment durations, and patient populations. The review underscores the need for standardized methodologies and additional large-scale, high-quality RCTs to more clearly define the role of probiotics in infection prevention within hospital settings [141].

The systematic review and meta-analysis of Asaadi et al. [142] included six randomized controlled trials involving a total of 391 patients with traumatic brain injury (TBI) to assess the effects of probiotic supplementation on clinical outcomes. The findings revealed that, in patients over the age of fifty, probiotics were associated with a statistically significant improvement in Glasgow Coma Scale (GCS) scores. However, no significant changes were observed in organ dysfunction (SOFA) or illness severity (APACHE II) scores. Notably, probiotic supplementation significantly reduced both the incidence of nosocomial infections and mortality. In contrast, it had no apparent effect on ICU admission rates or length of hospital stay. The authors propose that probiotics, by modulating the gut microbiota and supporting immune balance, may represent a promising adjunctive strategy in TBI treatment. Nonetheless, they caution that the current body of evidence is limited, and larger, well-designed clinical trials are needed to confirm these preliminary findings.

The beneficial effects of probiotics are supported by numerous reviews and meta-analyses, but there are also numerous clinical case reports demonstrating a direct link between probiotic use and bloodstream infections (Table 2).

### 4.4. Conflicting Conclusions Regarding the Efficacy and Safety of Probiotics

Recent systematic reviews and meta-analyses acknowledge the presence of clinical heterogeneity among cohorts. Variations in inclusion criteria are a major reason why existing studies show conflicting conclusions regarding the efficacy and safety of probiotics in critically ill patients. They include patients with sepsis, trauma, pancreatitis, respiratory failure, surgical complications, and more. Outcomes associated with probiotics can vary widely based on disease type, degree of immune suppression, baseline risk of infection, and presence of gastrointestinal dysfunction. Some meta-analyses and systematic reviews have highlighted those trials enrolling different ICU populations (medical vs. surgical, trauma vs. sepsis) report different efficacy signals for probiotics on infection reduction, particularly VAP. Subgroup analyses indicate that the benefits of probiotics (reduced ICU-acquired infections or VAP) may be more pronounced in certain patient groups, but neutral in others. For instance, some evidence points to greater benefits with longer probiotic use, higher dosages, or in patients with a specific diagnosis, but these findings are not consistent across all studies due to inclusion criteria [146,147].

Besides cohort selection, the probiotic species/strain, formulations (single vs. multi-strain), dosing, and duration of therapy also vary greatly, contributing to heterogeneity in results across studies [147].

There is also an impact of background therapies. Extensive antibiotic use, differences in nutrition (enteral vs. parenteral feeding), and other concurrent ICU interventions strongly affect both the baseline microbiome and the probability of a probiotic exerting a measurable effect. These factors are rarely standardized or well-documented in studies [148].

Older and smaller trials, or those with high risk of bias (poor randomization, lack of blinding, incomplete data), may report larger apparent benefits versus more rigorous, recent studies. For example, sensitivity analyses often show that positive results largely disappear when high risk of bias studies are excluded [149].

Another reason might be the use of different clinical endpoints (infection, ventilator-associated pneumonia, diarrhea, mortality), diagnostic criteria, and follow-up durations, causing heterogeneity in reported results. Additionally, endpoints such as “infection” or “sepsis” are variably defined across studies [148].

Careful attention to cohort selection, with targeted subgroup analyses, is essential to determine who might benefit or be harmed by probiotic therapy in critical illness.

## 5. Conclusions and Future Perspectives

Current human studies have established that ICU-related interventions, such as broad-spectrum antibiotics, mechanical ventilation, and parenteral nutrition, lead to significant microbiota dysbiosis, which correlates with worsened outcomes including infections and organ dysfunction [150,151]. Complementary animal models have elucidated mechanisms underlying these dysbiotic changes and their systemic effects, particularly highlighting the gut–organ axes that contribute to systemic inflammation and multi-organ failure in critical illness [152,153]. Despite sustained and valuable progress in this field, substantial gaps remain.

Future studies should address the problems of heterogeneous patient populations, variable therapeutic protocols and limited longitudinal data. The safety, efficacy, and optimal timing of microbiota-targeted therapies, including probiotics, prebiotics, and fecal microbiota transplantation should be validated through large-scale, randomized controlled trials. Moreover, while most current studies have focused on bacterial communities, the role of other microbiota components such as fungi and viruses remains underexplored and should be a focus of future research.

## Figures and Tables

**Table 1:** Nutraceutical agents and their main effects.

	Nutraceutical Composition	Role	References
**Probiotics**	Lactobacillus rhamnosus GG	-regulate the composition of the gut microbiota -improve epithelial barrier function -synergistically combined benefits with drugs -promising candidate management of ulcerative colitis	[126,127,128,129]
	Lactiplantibacillus plantarum	-reduces constipation -strengthens the mucosal-intestinal barrier -increases serotonin production -reduces intestinal oxidative stress	[130]
	Bifidobacterium bifidum BGN4 and Bifidobacterium longum BORI	-increased brain-derived neurotrophic factor -improves cognition and memory in the mouse model of Alzheimer’s disease -decreased neuroinflammatory response	[131]
	Akkermansia muciniphila and Bifidobacterium bifidum	-suppressed intestinal FXR expression -modulated the gut microbiota -improved intestinal mucosal permeability -protected against high-fat diets-induced non-alcoholic fatty liver disease formation	[132]
	Bacillus clausii spores	-inhibited the TXNIP/NLPR3 cascade and alleviated inflammation in colitis -influenced the Nrf2/HO-1 pathway and reduced oxidative stress in colitis -decreased the abundance of Firmicutes and Bacteroidetes in colitis -regulated caspase-3, Bax, and Bcl-2 levels and decreased colonic apoptosis	[133]
**Prebiotics**	Inulin	-shows potential as a treatment to decrease serum uric acid in renal failure patients, possibly through the microbial degradation of uric acid in the gut -reduces diet-induced barrier dysfunction and stimulates the expression of Paneth cell antimicrobial peptides	[134,135]
	Fructooligosaccharides (FOS) and Galactooligosaccharides (GOS)	-modulated the gut microbiota -modulated IRS/PI3K/AKT signaling pathway -reduced neuroinflammation facilitated neuroplasticity, resulting in better spatial learning and memory	[136]
	Oat β-glucan	-significantly alleviated metabolic dysfunction-associated steatohepatitis -increased Akkermansia, Ileibacterium, and Muribaculaceae -decreased Romboutsia and Enterococcus	[137]

**Table 2:** Probiotics—clinical use and complications.

Probiotics	Clinical Use	Complications	References
*Lactobacillus rhamnosus* and Bifidobacterium infantis	To prevent necrotizing enterocolitis	Sepsis with *Lactobacillus rhamnosus*	[143]
*Saccharomyces cerevisiae*	Adjunctive therapy in COVID-19, respiratory failure, ARDS and renal failure ICU admitted patients, treated with antibiotics and vasoactive amines	Bloodstream infection with Saccharomyces	[144]
*Lactobacillus* spp.	Adjunctive therapy in acute respiratory and haemorrhagic shock patients admitted to the ICU	Bloodstream infection with *Lactobacillus* spp., identified as *L. rhamnosus*	[145]
	Adjunctive therapy for acute respiratory failure ICU patients	Bloodstream infection with *Lactobacillus* spp., identified as *L. rhamnosus*	

## Data Availability

No new data were created or analyzed in this study. Data sharing is not applicable to this article.

## Abbreviations

The following abbreviations are used in this manuscript:

5-HT	5-hydroxytryptamine
AhR	Aryl hydrocarbon receptor
ARDS	Acute respiratory distress syndrome
BA	Bile acids
CDI	Clostridoides difficile infection
CFU	Colony forming unit
EHEC	Enterohemorrhagic *E. coli*
EN	Enteral nutrition
END	Enteral nutrition-related diarrhea
ETEC	Enterotoxigenic *E. coli*
FGF	Fibroblast growth factor
FXR	Farnesoid X receptor
GCS	Glasgow coma scale
GI	Gastrointestinal
GPBAR1	G-protein-coupled bile receptor 1
HAI	Hospital-acquired infections
ICU	Intensive Care Unit
IgA	Immunoglobulin A
KP	Kynurenine
LPS	Lipopolysaccharide
MLCK	Myosin light chain kinase
NAFLD	Non-alcoholic fatty liver disease
NF-kB	Nuclear factor kappa B
NGS	Next-generation sequencing
NMR	Nuclear magnetic resonance
NSAIDs	Non-steroidal anti-inflammatory drugs
